# Integrative Taxonomy Reveals Hidden Diversity in the *Aloina catillum* Complex (Pottiaceae, Bryophyta)

**DOI:** 10.3390/plants13030445

**Published:** 2024-02-02

**Authors:** María J. Cano, Juan A. Jiménez, Mónica Martínez, Lars Hedenäs, M. Teresa Gallego, Omar Rodríguez, Juan Guerra

**Affiliations:** 1 Departamento de Biología Vegetal (Botánica), Facultad de Biología, Universidad de Murcia, Campus de Espinardo, 30100 Murcia, Spain; jajimene@um.es (J.A.J.); monica.martinez11@um.es (M.M.); mgallego@um.es (M.T.G.); omar.rodrigueza@um.es (O.R.); jguerra@um.es (J.G.); 2 Department of Botany, Swedish Museum of Natural History, P.O. Box 50007, 104 05 Stockholm, Sweden; lars.hedenas@nrm.se

**Keywords:** ASAP, *atp*B-*rbc*L, bryophytes, cryptic diversity, ITS, NeighborNet split network, phylogenetic analysis, South America, taxonomy, *trn*G, *trn*L-F

## Abstract

*Aloina catillum* is a variable moss typical of xerophytic environments in the Neotropics, characterized against other closely allied *Aloina* species with well-differentiated leaf border by its setae twisted to the left throughout. In order to clarify its variability and its relationships with the allied species with differentiated leaf border *A. brevirostris*, *A. obliquifolia*, and *A. rigida*, we performed an integrative study including sequence data from four markers (nuclear ITS, plastid *atp*B-*rbc*L, *trn*G, *trn*L-F), morphometry, and species assembling by automatic partitioning (ASAP) algorithm. Our data suggest that *A. catillum* consists of at least three species: *A. calceolifolia* (an earlier name for *A*. *catillum*), and two species described here as a new, *A. bracteata* sp. nov. and *A. limbata* sp. nov. This latter species includes the specimens previously identified as *A. obliquifolia* from South America. Additionally, some morphological and molecular variability was also detected in *A. limbata*, but was not consistent enough to be recognized taxonomically. The study supports the presence of *A. brevirostris* in the Neotropics and *A. rigida* is tentatively excluded from South America. Full descriptions of the *A. catillum* s.l. species and a diagnostic key to this complex in South America are provided.

## 1. Introduction

The incorporation of molecular phylogenetic studies into taxonomy has greatly improved our understanding of relationships within bryophytes and has uncovered extensive hidden diversity in groups with morphological characters that are difficult to interpret. Cryptic species is a common and increasingly used term that refers to taxa that cannot readily be distinguished morphologically, yet evidence indicates they are on different evolutionary trajectories [1,2]. In addition, species with minor morphological differences found after reinvestigations are usually named by terms such as semi-cryptic or pseudo-cryptic [3].

With the increasing use of molecular approaches in recent decades, many bryophyte studies have been published on such variation (e.g., [4,5,6,7,8,9]), which represents a significant portion of undiscovered biodiversity. However, many of these studies come from northern temperate regions and very few treat cryptic or pseudo-cryptic species from the Southern Hemisphere [10,11]. Discoveries of cryptic or pseudo-cryptic diversity may alter the assumed distribution of diversity and endemism, which in turn may change our understanding of evolutionary processes and could have profound implications for biogeography and conservation planning [1].

**Figure 1:**
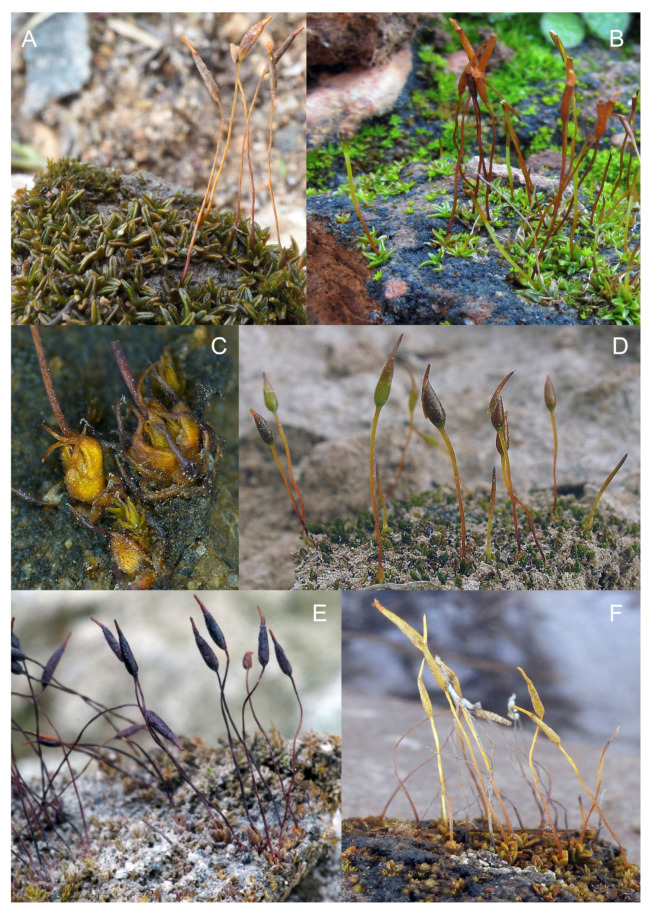
Habit of plants from the *Aloina catillum* complex and allied species. (**A**) *Aloina brevirostris* (Cano et al. 3403a, MUB 27728). (**B**) *Aloina rigida* (Cano 4843, MUB 28934). (**C**) *Aloina obliquifolia* (*A. limbata*) (Cano et al. 7649, MUB 49149). (**D**) *Aloina catillum* s.l. (*A. bracteata*) (Cano et al. 4113, MUB 29597). (**E**) *Aloina catillum* s.l. (*A. limbata*) (Cano 5065, MUB 32542). (**F**) *Aloina catillum* s.l. *A. calceolifolia*) (Cano and Alonso 8345, MUB 56422). Photos: (**A**,**C**–**F** Carlos Aedo; **B** María J. Cano.

*Aloina* Kindb. is a small genus of the moss family Pottiaceae adapted to xerophytic environments [12]. The genus is well characterized morphologically by its leaves, usually differentiated into a sheathing base and a concave blade with the margins incurved and ventral chlorophyllous filaments that extend over the costa and the lamina surface. However, the species level identification of some of their complexes such as *A. ambigua* (Bruch & Schimp.) Limpr.-*A. aloides* (W.D.J. Koch ex Schultz) Kindb., or *A. rigida* (Hedw.) Limpr.-*A. obliquifolia* (Müll. Hal.) Broth. is well known to be problematic. Thus, there still exist disagreements regarding how many species to recognize in this genus, according to different authors [13,14,15]. This genus is less frequent in the Southern Hemisphere than in the Northern Hemisphere where, however, it appears to be strongly diversified in number of species and endemism. In South America, seven species have been reported. One group of species (*A. bifrons* (De Not.) Delgad., *A. roseae* (R.S. Williams) Delgad., and *A. scindulosa* M.J. Cano, J.A. Jiménez & M.T. Gallego is characterized by unbordered leaves, with a long, hyaline hair-point [12,13]. The other group, formed by *A. brevirostris* (Hook. & Grev.) Kindb., *A. catillum* (Müll. Hal.) Broth., *A. obliquifolia*, and *A. rigida*, is characterized by leaves bordered by elongate or oblate, thin-walled, hyaline cells disposed in several columns. *Aloina brevirostris* is known from a single locality in the Magallanes Region of southern Chile [16], and *A. catillum*, *A. obliquifolia*, and *A. rigida* have been reported from a few localities in dry areas of the tropical Andes [17,18,19] (Figure 1). Two poorly known species, due to the unavailability of type material [18], *A. recurvipatula* (Müll. Hal.) Broth. and *A. sedifolia* (Müll. Hal) Broth., described from Argentina, seem to exhibit a differentiated border, according to their protologues [20,21], and could be related with this group of species [12].

*Aloina catillum* is a poorly known species described by Müller [20] from a single Argentinian specimen. More recently, this species has been reported from a few localities in the tropical Andes of Chile, Ecuador, and Peru [13,19]. Delgadillo [13], in his world revision of *Aloina*, tentatively recognized this species, hoping that more collections were available, because he only could study the type material and one additional specimen. He characterized *A. catillum* by its suborbicular leaves, with marginal cells differentiated and the long ovoid-cylindrical capsules. This author also indicated that the cladautoicous, paroicous, or dioicous condition and the extremely broad costa may eventually be found to be taxonomically useful characters. Regarding sporophytic differentiation, Delgadillo [13] stated that in *A. catillum*, the seta turns to the left above, unlike the other species of *Aloina* with leaves bordered, such as *A. rigida* (including *A. obliquifolia*) or *A. brevirostris*, where it is dextrorse above. He also noted that most features of *A. catillum* show considerable overlap with those of *A. rigida*. Thus, *A. calceolifolia* (Spruce ex Mitt.) Broth., treated as a synonym of *A. rigida* by Delgadillo [13], had some leaves as broadly lingulate as some of those in *A. catillum*, and the seta, at least in the type specimens of this taxon, twisted to the left throughout. Unlike the Neotropical *A. catillum*, *A. brevirostris* exhibits a bipolar distribution [22]. *Aloina rigida* is widespread in the Northern Hemisphere, but not reaching the Arctic, with a few records in South America and tropical Africa [13,15]. *Aloina obliquifolia* is easily distinguished by the leaf costa excurrent as a stiff awn or mucro. It was described from China [23] as *Barbula obliquifolia* Müll. Hal., and later reported from Japan [24]. *Aloina rigida* var. *mucronulata* (Bruch & Schimp.) Limpr. was considered to be conspecific with *A. obliquifolia*, extending the distribution of this taxon to Europe [15]. Subsequently, it was reported from Ecuador and Peru in the tropical Andes [19]. There is another taxon with leaves bordered that is very close to *A. obliquifolia*, known only from two collections from China, *A. cornifolia* Delgad., which is distinguished mainly by the presence of a strongly cucullate leaf apex, with the mucro developing from the dorsal side of a hood-like apex [13,25]. Despite the existence of a global monograph of the genus [13] and a taxonomic revision in the Mediterranean area [15], there is no comprehensive molecular phylogeny of this genus of the Pottiaceae and only a few *Aloina* sequences used as outgroups have been published (e.g., [26,27]). Therefore, the current morphology-based species concept in this genus has not been tested by molecular evidence.

In our ongoing study of the Pottiaceae in South America, we have had the opportunity to collect new material belonging to the genus *Aloina* in Andean dry areas. Most specimens were dioicous without sporophytes, making the identification quite difficult. In addition, we found a large morphological variation in the gametophytic and sporophytic characters in the specimens identified as *A. catillum*, which may be due to overlooked diversity within the genus. To clarify this, in the framework of our ongoing and integrative taxonomic approach to the genus *Aloina*, a study of *A. catillum* and related species was performed. Based on preliminary morphological identification of the specimens from South America, we hypothesized that *A. catillum* s.l. could represent several distinct evolutionary entities. Our aims were: (1) To test if the molecular topology is in accordance with the current morphology-based species concept in *A. catillum* s. str. and related species; (2) To quantify the morphological variation in *A. catillum* s.l. and to determine if this variation could be correlated with discrete taxonomic entities.

## 2. Materials and Methods

### 2.1. Study Species and Material

A total of 37 specimens currently referable to *Aloina catillum* were selected for the molecular and morphological evaluation in order to span its morphological variability and geographical range. In addition, eleven specimens of *A. obliquifolia*, nine of *A. brevirostris*, and six of *A. rigida* were included on the basis of their well-differentiated leaf border. With the intention to assess the position of *A. catillum* s.l. and related species within the genus, three additional species, *A. aloides* (one specimen), *A. ambigua* (one specimen), and *A. bifrons* (two specimens), were included. Moreover, three representatives of other genera of the subfamily Pottioideae (*Aloinella* Cardot, *Crossidium* Jur., and *Erythrophyllopsis* Broth.) were selected to form the outgroup of the molecular analysis based on previous phylogenetic studies [27,28]. Most specimens were collected during several fieldwork visits by the authors in Argentina, Bolivia, Cape Verde, Cyprus, Ecuador, Greece (Crete), Peru, Spain, and Venezuela. Full details on the fieldwork localities are available at the website of the group (www.pottiaceae.com; accessed on 15 September 2023). In addition, herbarium material from CAS, MUB, UBC, the private herbarium of T.L. Blockeel, and type material of the taxa currently attributed to *Aloina* deposited at E, H, NY, PC, and S were examined. Details of the plant material, including voucher information, accession provenance, and GenBank accession numbers, are provided in Appendix A.

### 2.2. DNA Extraction, Amplification, and Sequencing

Plant tissue from the distal portions of gametophore shoots was isolated from herbarium specimens or recent collections. Whole genome DNA was then extracted, either using a modified version of the CTAB [29] or the protocol for extraction by Suzuki et al. [30], and stored at −20 °C until the polymerase chain reaction (PCR) was carried out. We selected four loci; three from the chloroplast genome, the *atp*B-*rbc*L intergenic spacer region (*atp*B-*rbc*L), the *trn*G_UCC_ G2 intron (*trn*G), and the *trn*L_UAA_ exon *trn*F_GAA_ region (*trn*L-F), as well as the nuclear internal transcribed spacers 1 and 2 (ITS1-5.8S-ITS2). The ITS1 and ITS2 were either amplified and sequenced separately or in a single amplification. All loci have been shown to be useful for phylogenetic reconstruction in the Pottiaceae [27,28,31]. Although paralogous ITS haplotypes have been reported in this family [32,33], no specimens with different paralogues were found in *Aloina*. Therefore, the revealed limited ITS variation was interpreted as being among homologous haplotypes.

The primer pairs used for each locus were *atp*B-*1/rbc*L-*1* [34], *trnG*-F/*trn*G-R [35], *trn*C*/trn*F [36], ITS5-bryo/ITS4-bryo [37], ITS1-F/ITS1-R [38], and seqITS2 [39]. Amplification reactions were performed using an Eppendorf Mastercycler in a 25 μL volume containing 10 μL Supreme NZYTaq II 2x Green Master Mix (Nzytech, Lisbon, Portugal), 2 μL of each primer (10 µm), and 1 μL of the DNA extract. Thermocycling conditions for *atp*B–*rbc*L, *trn*G, and *trn*L–F were 95 °C for 4 min linked to 35 cycles at 94 °C for 30 s, 52 °C for 30 s, and 72 °C for 7 min, with a final extension of 72 °C for 7 min. The amplification cycle for nrITS was 95 °C for 4 min linked to 35 cycles at 94 °C for 1 min, 55 °C for 1 min, and 72 °C for 1 min and 30 s, with a final extension of 72 °C for 7 min. PCR products were visualized on a 1% agarose gel. Successful amplifications were purified using the GenElute PCR Clean-Up kit (Sigma-Aldrich, St. Louis, MO, USA), and sequenced at Macrogen Spain (Madrid, Spain). Nucleotide sequence contigs were edited and assembled for each DNA region using Geneious 9.1.8 [40]. Consensus sequences were aligned using default parameters of MUSCLE [41] implemented in Geneious with subsequent manual adjustments. Regions of partially incomplete data at the beginning and end of the sequences were identified and excluded from subsequent analyses. Insertions and deletions (indels) were coded using SeqState v.1.4.1 [42] using a simple coding model as suggested by Simmons and Ochoterena [43]. The indels provided additional phylogenetic evidence and we present the analyses with these included. Each gene partition was tested for the best-fit substitution model using jModelTest v.2.1.7 [44] under the Akaike information criterion (AIC). The selected models were TPM3uf + I [45] for *trn*G, HKY + I [46] for *trn*L–F, TIM3ef + I + G [47] for nrITS, and TPM1uf + I + G [45] for *atp*B-*rbc*L.

### 2.3. Phylogenetic Analysis

Phylogenetic relationships were estimated using both maximum likelihood (ML) and Bayesian inference (BI). Analyses were performed separately on each dataset and the plastids were combined afterwards. To check for incongruence among the plastid datasets and between plastid versus ITS partitions, phylogeny reconstructions under ML and BI were visually compared. Because some incongruence between the nuclear and plastid markers for the studied taxa was found, we analyzed ITS and plastid markers separately. Maximum likelihood analyses were performed with RAxML [48] through the graphical front-end raxmlGUI v.2.0 [49]. A rapid bootstrap option with 1000 replicates and search for the best-scoring ML tree were conducted under the GTRCAT model for the concatenated and individual datasets. Nodes with bootstrap (BS) values of 70–89% were treated as moderately, and of 90–100% as well supported.

Bayesian inference was performed using MrBayes v.3.2.6 [50], running a partitioned analysis and specifying a substitution model for each block. The data were analyzed using Markov chain Monte Carlo (MCMC), running two parallel analyses with four chains each for 30 million generations, sampling trees and parameters every 1000 generations. Chain convergence and stationarity were checked in Tracer v.1.7 [51], making sure the average standard deviation of split frequencies remained below 0.01. Twenty-five percent of the trees were discarded as burn-in, and a 50% majority-rule consensus tree was constructed. The resulting trees for both ML and BI analyses were visualized and partially edited in FigTree v.1.4.4 [52]. Posterior probability (PP) of 0.95–1.00 was considered to be strong support.

Since the topologies inferred from the nuclear and plastid markers may imply reticulate evolution or incomplete lineage sorting, we used the NeighborNet (NN) method as implemented in SplitsTree v.4.16.2 [53] to visualize affinities between lineages within the *Aloina catillum* complex and the outgroups included.

Assemble Species by Automatic Partitioning (ASAP) [54] was applied to delimit molecular entities that may be considered a hypothesis for species within the *Aloina catillum* complex. This method proposes species partitions using a hierarchical clustering algorithm based on pairwise genetic distances. The analyses were performed on the ASAP web server (https://bioinfo.mnhn.fr/abi/public/asap/; accessed on 12 June 2023), running the concatenated chloroplast and ITS datasets separately, with default parameters. ASAP delimitation was defined evaluating both the partitions with best and the second-best asap-score [54]. 

### 2.4. Morphological-Anatomical Analysis

After the molecular relationships were established within *Aloina catillum* s.l., the morphology of the specimens belonging to the distinguished molecular entities of this complex was examined. In addition, the specimens of *A. brevirostris*, *A. obliquifolia*, and *A. rigida*, all of them with a distinct leaf border, were also included in the analysis. Microscopic examinations and measurements were taken with an Olympus-BX41 light microscope (Olympus, Tokyo, Japan), while microphotographs were obtained with a Jenoptik ProgRes C7 camera (Jenoptik, Jena, Germany) mounted on this microscope. Specimens were examined in 2% potassium hydroxide. 

**Figure 2:**
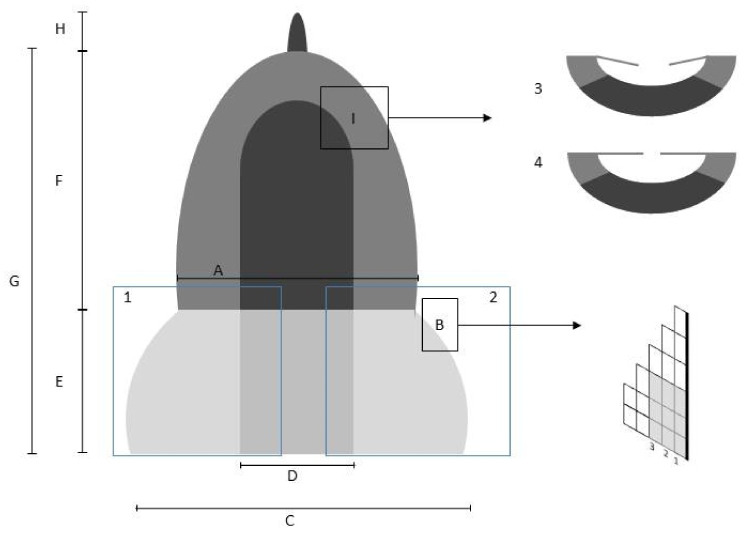
A schematic vegetative leaf of *Aloina*, indicating the parts measured or assessed in the morphological study: (A) Cross section at mid-leaf; (B) Number of columns at the leaf border; (C) Leaf width; (D) Costa width; (E) Sheathing base with two shoulders highlighted as 1 and 2; (F) Limb; (G) Leaf length measure; (H) Mucro/awn length; (I) Leaf margins showing 3, margins bent towards the costa in both sides, and 4, margins parallel to the costa in both sides.

We studied morphological-anatomical quantitative and qualitative characters, including several ratios, which could be used for the identification of the species. Preference was given to those characters which Delgadillo [13] and Gallego et al. [15] considered important for taxa distinction, as well as those representative of the morphological variability among the studied specimens. To avoid developmental deviations, descriptions and measures were made from upper vegetative leaves (Figure 2). Cross-sections of the leaves were made at mid-leaf. The number of cell columns at the leaf border was scored in the upper part of the sheathing base. Leaf width was measured at the base. In addition, the width and length of the apical cells of ventral surface costal filaments were measured in cross-section. Three shoots were selected at random from each collection, and measurements were performed on three randomly selected leaves. Measurements of sporophytic traits were performed on 1–3 capsules depending on their availability for each specimen. To represent the variability of each character, boxplots containing medians and percentiles were performed. The analyses were implemented using the STATISTICA v. 10 package (StatSoft Inc., Tulsa, OK, USA).

## 3. Results

### 3.1. Molecular Analyses

In total, the analysis included 231 sequences, of which 212 (91.8%) were newly generated for this study; 19 additional sequences, that were sequenced previously in other phylogenetic studies [26,27,28,55], were downloaded from GenBank. Summary characteristics of each dataset are presented in Table 1. 

**Figure 3:**
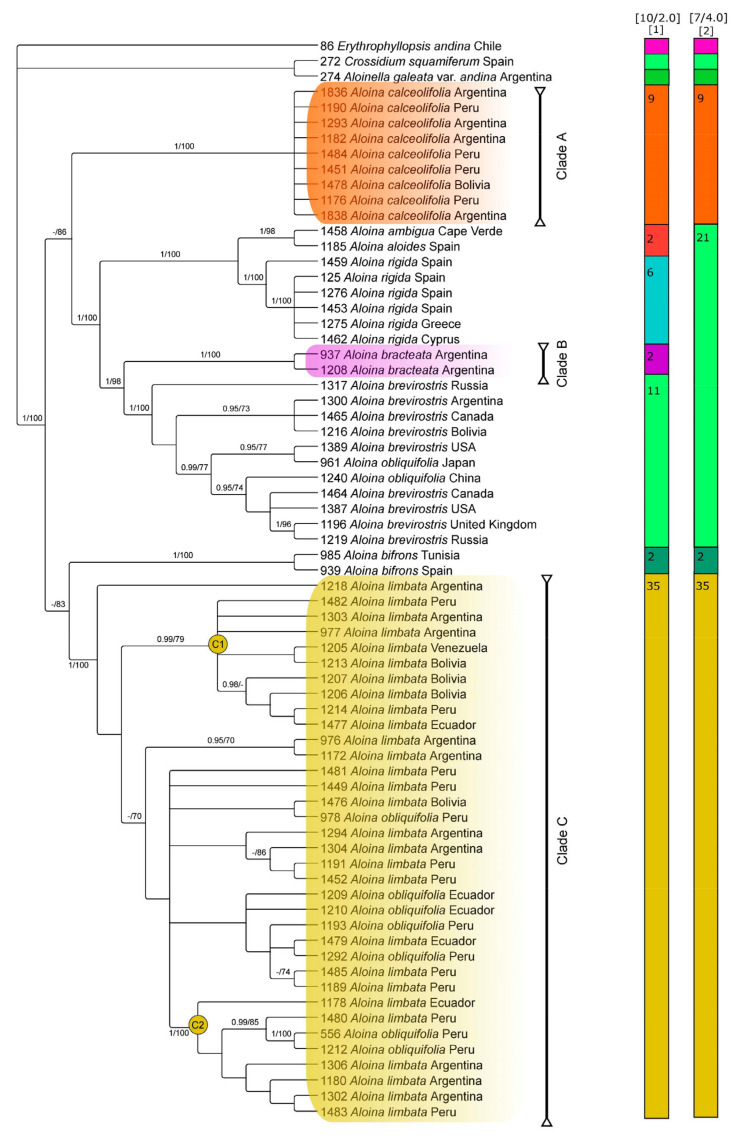
Bayesian tree of the *Aloina catillum* complex inferred from the ITS dataset. Bayesian posterior probabilities (PP), followed by maximum likelihood bootstrap values (BS), are shown above the branches. Support values of BS < 70 and PP < 0.95 are not shown. The clades that include species considered in *A. catillum* s.l. in this study are labeled A to C and are referred to in the text. Letter and numbers C1–C2 indicate nodes discussed in the text. Colored bars on the right show the putative species inferred by ASAP.

**Figure 4:**
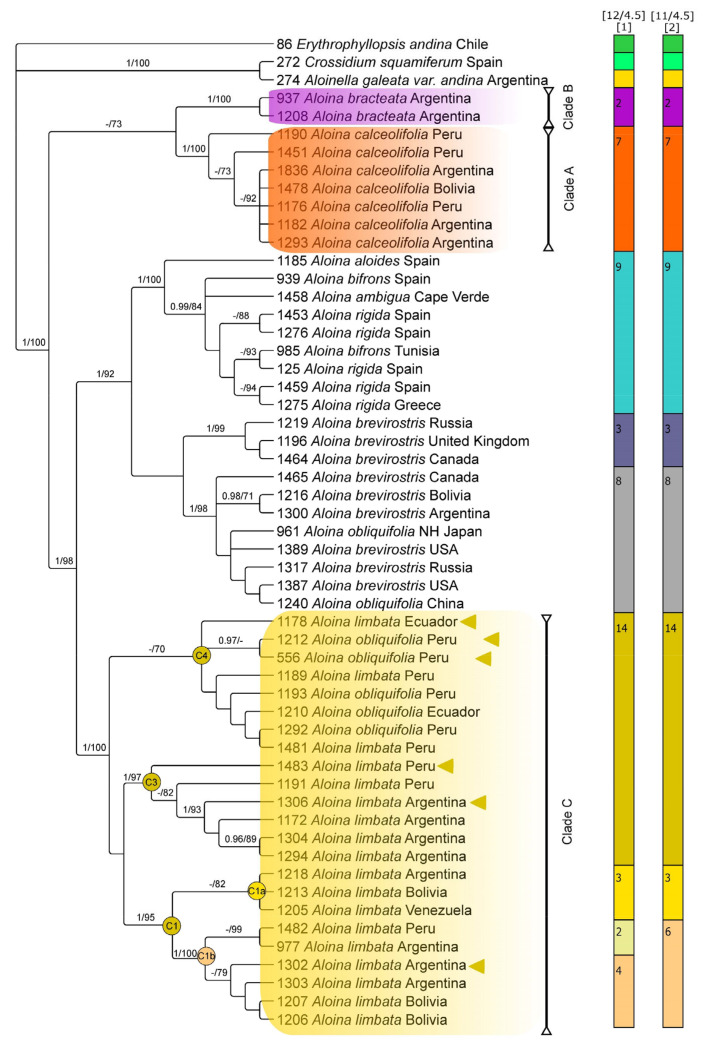
Bayesian tree of the *Aloina catillum* complex inferred from the combined three-plastid dataset. Bayesian posterior probabilities (PP), followed by maximum likelihood bootstrap values (BS), are shown above the branches. Support values of BS < 70 and PP < 0.95 are not shown. The clades that include species considered in *A. catillum* s.l. in this study are labeled A to C and are referred to in the text. Letters and numbers C1, C1a, C1b, C3, and C4 indicate nodes discussed in the text. Yellow triangles in the clades C1b, C3, and C4 indicate the specimens of *A. catillum* which were grouped in clade C2 in the ITS analysis. Colored bars on the right show the putative species inferred by ASAP.

The ML and BI analyses of each individual marker resulted in largely congruent trees. Therefore, only the Bayesian topologies are shown here (Figure 3 and Figure 4), with ML and BI support values added where applicable. The plastid and nuclear datasets were not combined due to some topology incongruences revealed when the plastid and nuclear consensus tree were compared. 

**Figure 5:**
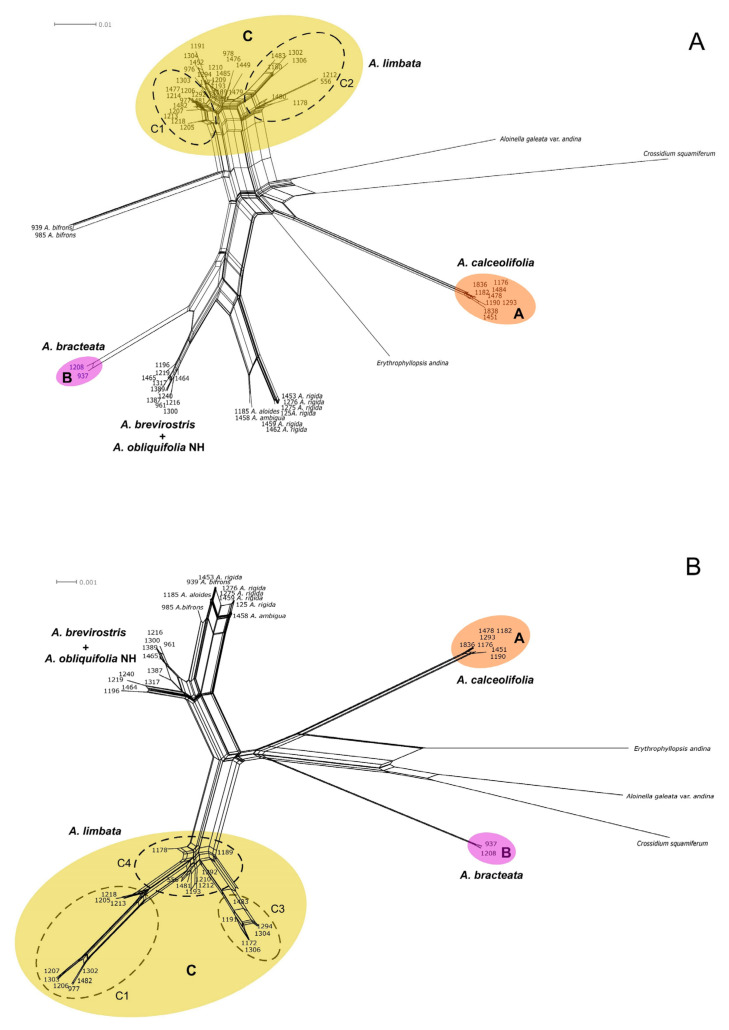
Neighbor-Net network built using SplitsTree, originating from the nr ITS (**A**) and plastid (**B**) datasets. Colors indicate revealed lineages of the *Aloina catillum* complex.

The topologies of trees inferred from the combined plastid, and nuclear data support that the genus *Aloina* is monophyletic. On the other hand, plastid and ITS data, as well as NeighborNet split networks (Figure 5), support the recognition of three lineages within *Aloina catillum* s.l. These are labeled clade A, which includes accessions identified previously as *A. catillum* or *A. rigida*; clade B, which includes two distinctive samples of *Aloina;* and clade C, which includes the majority of the accessions previously determined as *A. catillum* and the specimens identified as *Aloina obliquifolia* from South America (Figure 3, Figure 4 and Figure 5).

In the phylogenetic tree derived from ITS sequences (Figure 3), the clade A (PP = 1, BS = 100) is moderately supported by ML (BS = 86) as sister to a large clade strongly supported (PP = 1; BS = 100), which includes *A. aloides*, *A. ambigua*, *A. rigida*, clade B, *A. brevirostris*, and part of the specimens of *A. obliquifolia*. *Aloina ambigua*, and *A. aloides* (PP = 1, BS = 98) are resolved sister to a well-supported clade of *A. rigida* (PP = 1, BS = 100). In turn, this latter clade is sister to a clade well supported (PP = 1; BS = 98), including clade B (PP = 1, BS = 100) and a clade with the accessions of *A. obliquifolia* from Northern Hemisphere nested with the accessions of *A. brevirostris* (PP = 1, BS = 100). 

Clade C comprises most of the specimens identified as *A. catillum* and the South American specimens identified as *A. obliquifolia* (PP = 1, BS = 100). This clade is moderately supported as sister to *A. bifrons* by ML (BS = 83). In clade C, two well-supported subclades can be distinguished: C1 (PP = 0.99, BS = 79), and C2 (PP = 1, BS = 100). 

The phylogenetic tree derived from combined plastid sequences (Figure 4) shows a moderately supported clade (BS = 73) with the accessions included in clade A sister to clade B, both with maximal support. The remaining accessions of *Aloina* were included in a well-supported clade (PP = 1, BS = 98). Within this latter clade, two well-supported subclades can be identified: (1) A clade formed by all accessions of *A. brevirostris* plus *A. obliquifolia* from the Northern Hemisphere, sister to the accessions of *A. aloides*, *A. ambigua*, *A. bifrons*, and *A. rigida* (PP = 1, BS = 92); (2) The clade C with the accessions of *A. catillum* and the South American specimens identified as *A. obliquifolia* (PP = 1, BS = 100). In this latter clade, the subclade C1 (PP = 1, BS = 95), also revealed in ITS, is supported with two subclades: C1a (BS = 82) and C1b (PP = 1, BS = 100), including two accessions (1218 and 1302) which were not placed in clade C1 in the ITS topology. The remaining accessions included were accommodated in two subclades, named C3 (PP = 1, BS = 97) and C4 (BS = 70). The lineage C2 (Figure 3), fully supported in the ITS tree, is resolved in the plastid tree in a ‘polytomy’ within the subclades C1b, C3, and C4.

The NN split networks (Figure 5) retrieved the same three clusters in the *A. catillum* complex as the trees based on the ITS and plastid markers. The clade C represents a highly divergent and complex structured dataset which splits into two supported subclades (C1 and C2) by ITS (Figure 5A) and three subclades (C1, C3, and C4) by plastid markers (Figure 5B).

The best ASAP for dividing the ITS dataset returned 10 partitions, which recognizes the three lineages in *A catillum* s.l. (Clades A, B, C) as separate putative species (Figure 3), in addition to the already existing species represented (with the particularity that *A. aloides* and *A. ambigua* are grouped and *A. brevirostris* includes the *A. obliquifolia* samples from the Northern Hemisphere). The second-best score suggested a division into seven species, with Clade B, *A. brevirostris* (including *A. obliquifolia* NH), *A. ambigua*, *A. aloides*, and *A. rigida* lumped. 

The best scoring for the plastid dataset proposed 12 species, which split *A. catillum* s.l. into six entities (clades A, B, and C, the latter split into four partitions). The second-best ASAP included an 11-species scheme, which split *A. catillum* s.l. into five entities: Clades A, B, and C, which split into three partitions (C1a, C1b, and C3 + C4) (Figure 4). In both cases, inconsistencies between described and delimited species were identified because *A. bifrons*, *A. ambigua*, *A. rigida*, and *A. aloides* sequences were merged.

### 3.2. Morphological Evaluation

**Figure 6:**
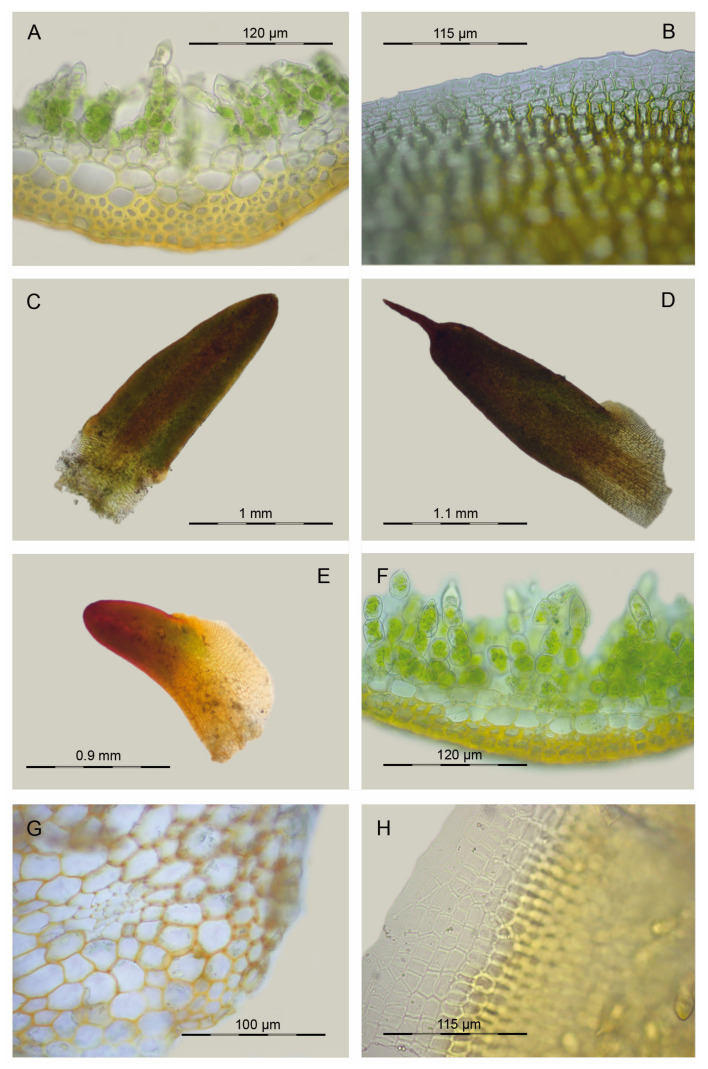
Distinctive morphological characters in *Aloina brevirostris*, *A. obliquifolia*, and *A. rigida*: (**A**) *A. rigida*, cross-section of the costa at the mid-leaf (Cano 11198, MUB 62244); (**B**) *A. rigida*, leaf border at the base (Cano 10198, MUB 62244); (**C**) *A. rigida*, vegetative leaf (Aedo 16324f, MUB 29388); (**D**) *A. obliquifolia*, vegetative leaf (Sato s.n., MUB 52915); (**E**) *A. brevirostris*, vegetative leaf (Cano et al. 4102, MUB 29586); (**F**) *A. brevirostris*, cross-section of the costa at the mid-leaf (Cano et al. 3403 (MUB 27728); (**G**) *A. brevirostris*, cross-section of a stem (Cano 7578, MUB 49074); (**H**) *A. brevirostris*, leaf border at the base (Cano 7578, MUB 49074). Photos: María J. Cano.

**Figure 7:**
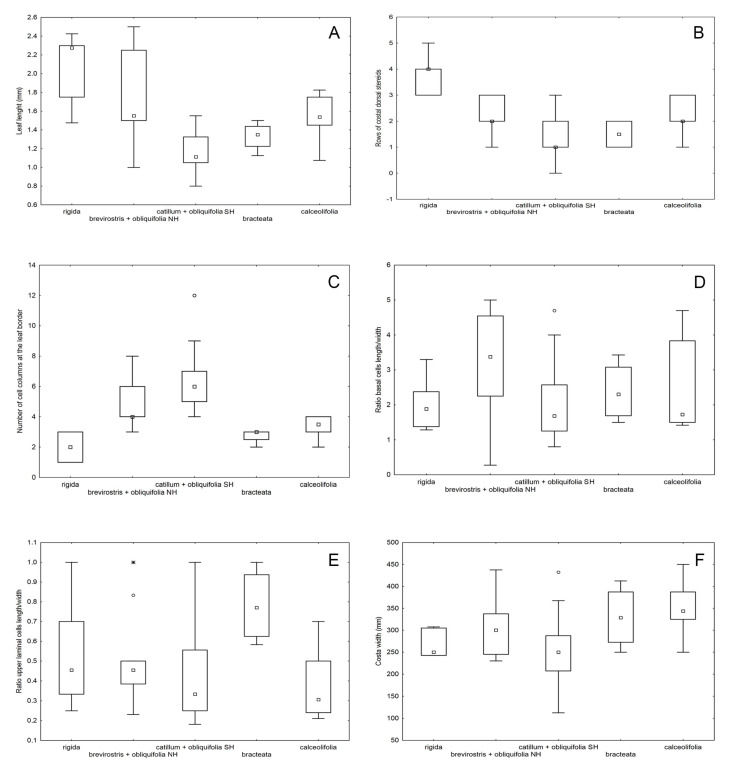
Boxplots showing the variation ranges of the most discriminate morphological gametophyte characters among the species studied: (**A**) Leaf length (mm); (**B**) Number of rows of dorsal stereids at the costa; (**C**) Number the columns at the leaf border; (**D**) Ratio of basal laminal cells length/width; (**E**) Ratio of upper laminal cell length/width; (**F**) Costa width (µm). The boxes represent the data from the 25th to 75th percentiles, with the median indicated within the box, and the lines above and below boxes are the total range. The small circles outside the bar represent “outlier” and the black stars are extremes. NH: Northern Hemisphere; SH: Southern Hemisphere. Note that “catillum + obliquifolia SH” is *A. limbata*.

Our morphological observations showed that the *Aloina catillum* complex can be distinguished from *A. rigida* and *A. brevirostris* by the seta twist direction: twisted to the left throughout in *A. catillum* s.l., and twisted to the left below and to the right above in *A. rigida* and *A. brevirostris*. Without sporophytes, *A. rigida* could be separated from these species that also have bordered leaves mainly by its higher number of dorsal stereid rows in the costa (Figure 6A and Figure 7B) and lower number of cell columns at the leaf border (Figure 6B and Figure 7C). On the other hand, *Aloina rigida* has longer leaves (Figure 6C and Figure 7A) than *A. catillum* s.l. 

We have not been able to study all previous records of *A. rigida* from the Neotropics, but the studied specimens were identified as belonging either to the *A. catillum* clade A or to *A. brevirostris*, representing the first reports of the latter from this geographic area. 

**Figure 8:**
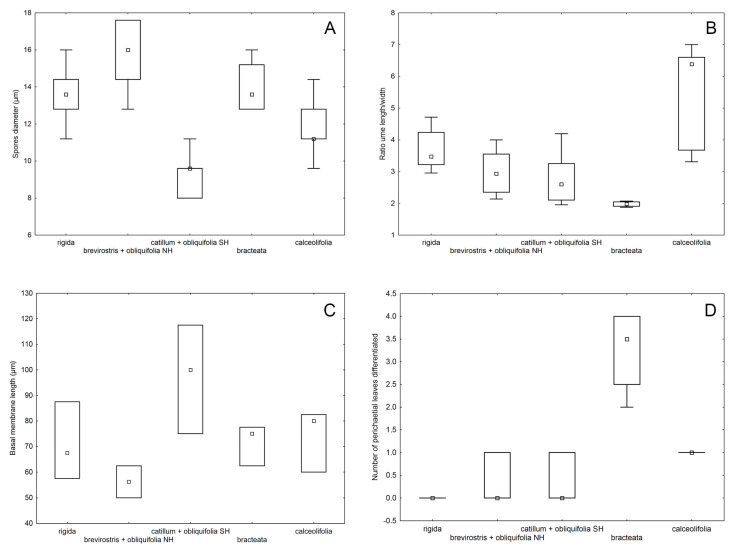
Boxplot showing variation ranges of the most discriminate morphological sporophyte characters among the species studied: (**A**) Spore diameter (µm); (**B**) Ratio urn length/width; (**C**) Height of the basal membrane of the peristome (µm); (**D**) Number of differentiated perichaetial leaves. The boxes represent the data from the 25th to 75th percentiles, with the median indicated within the box, and the lines above and below boxes are the total range. The small circles outside the bar represent “outlier” and the black stars are extremes. NH: Northern Hemisphere; SH: Southern Hemisphere. Note that “catillum + obliquifolia SH” is *A. limbata*.

**Figure 9:**
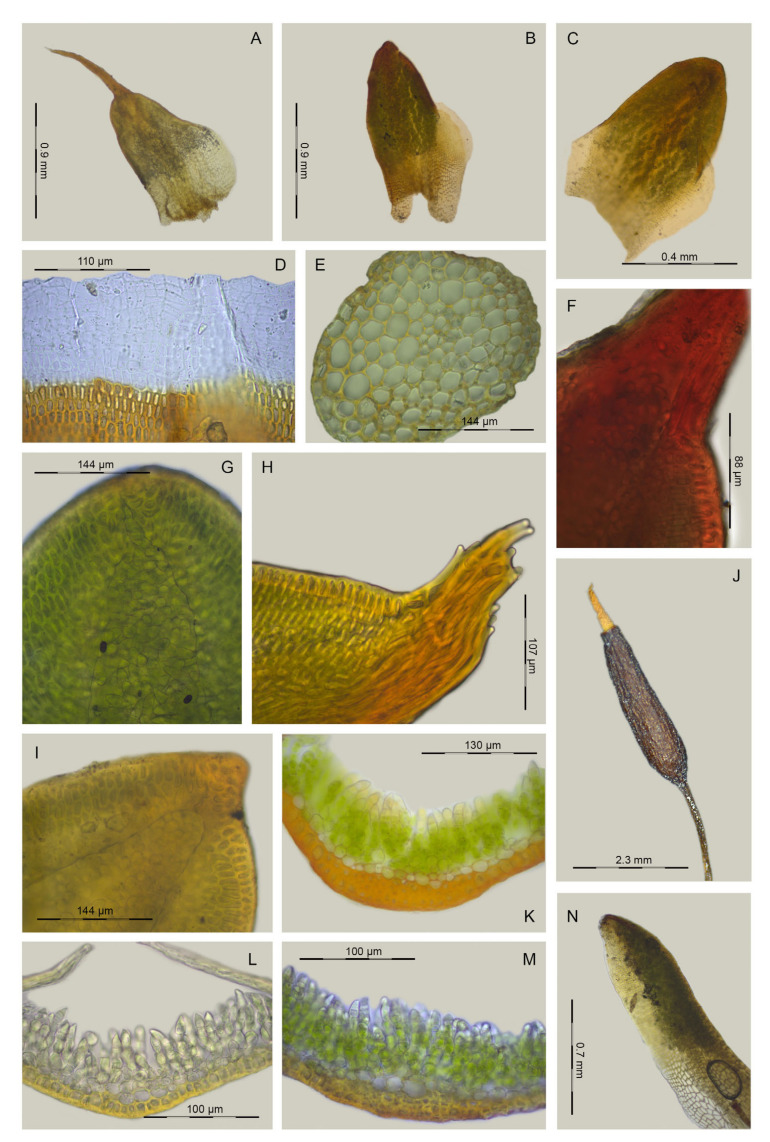
Distinctive morphological characters in *Aloina limbata*: (**A**–**C**) Vegetative leaves (**A**) Cano et al. 7649, MUB 49149; (**B**) Cano and Gallego 2903a, MUB 22314; (**C**) Cano and Jiménez 5168, MUB 32545; (**D**) Leaf border at the base (Cano et al. 7150, MUB 48588); (**E**) Cross-section of a stem (Cano and Gallego 2903a, MUB 22314); (**F**–**I**) Leaf apices (**F**) Cano et al. 2814b (MUB 59251); (**G**) Cano et al. 7075, MUB 48503; (**H**) Cano et al. 7150, MUB 48588; (**I**) Cano et al. 5076b (MUB 32548); (**J**) Urn and peristome (Cano et al. 5065, MUB 32542); (**K**–**M**) Cross-sections of the costa at mid-leaf (**K**) Cano et al. 4082, MUB 29585; (**L**) Cano et al. 7075, MUB 48503; (**M**) Cano et al. 7150, MUB 48588; (**N**) Perichaetial leaf (Cano and Gallego 2903a, MUB 22314). Photos: María J. Cano.

*Aloina brevirostris* could be separated from *A. catillum* clade C and *A. rigida* by its usually monoicous condition (synoicous or rhizoautoicous vs. dioicous) and its larger spores (Figure 8A). However, it could be more difficult to distinguish from *A. catillum* s.l. without sporophytes. In general, *A. brevirostris* usually has longer leaves (Figure 6E and Figure 7A), a higher ratio of basal laminal cell length/width (Figure 7D), and in some cases the central strand of the stem is slightly differentiated (Figure 6G), which is not the case in *A. catillum* complex (Figure 9E). Other characters suggested to be diagnostic in *A. brevirostris*, like the short conic or conic-rostrate operculum, have been found to be variable as already indicated by other authors [13,56].

**Figure 10:**
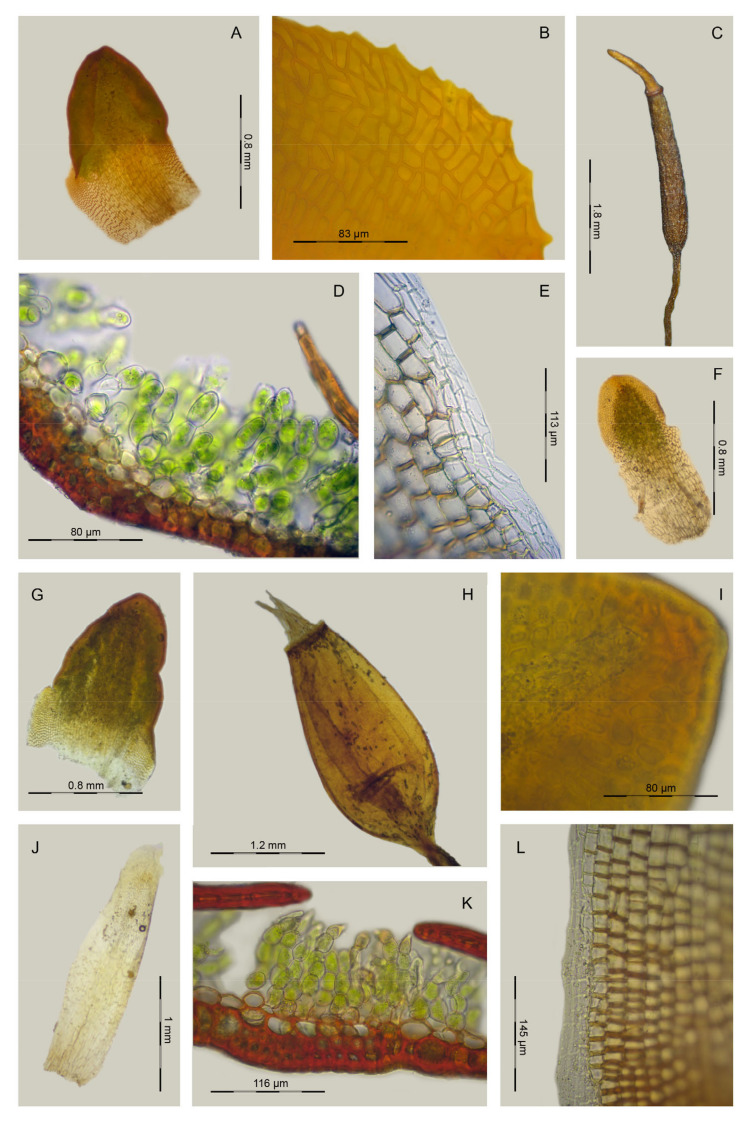
Distinctive morphological characters in *Aloina calceolifolia* and *A. bracteata*. *Aloina calceolifolia:* (**A**) Vegetative leaf (Cano and Alonso 8144, MUB 56217); (**B**) Leaf border at the middle (Cano 2353, MUB 20586); (**C**) Capsule (Cano 2353, MUB 20586); (**D**) Cross-section of the costa at mid-leaf (Cano and Alonso 8144, MUB 56217); (**E**) Leaf border at the base (Cano *2353*, MUB 20586); (**F**) Perichaetial leaf (Cano et al. 3383, MUB 27722). *Aloina bracteata*: (**G**) Vegetative leaf (Cano and Alonso 8409a, MUB 56491); (**H**) Urn and peristome (Cano et al. 4113, MUB 29597); (**I**) Leaf apex (Cano and Alonso 8409a, MUB 56491); (**J**) Perichaetial leaf (Cano and Alonso 8409a, MUB 56491); (**K**) Cross-section of the costa at mid-leaf (Cano and Alonso 8409a, MUB 56491); (**L**) Leaf border at the base (Cano and Alonso 8409a, MUB 56491). Photos: María J. Cano.

In *A. catillum* s.l., sporophytic characters such as spore diameter (Figure 8A), ratio urn length/width (Figure 8B), and height of the basal membrane (Figure 8C), have been revealed as distinctive in the separation of the species. However, these data should be considered with caution, since the number of sporophytes measured in the specimens of clade C was small. Among all studied features, the most significant gametophytic characters in the *A. catillum* complex were leaf length (Figure 7A and Figure 9A–C), ratio upper laminal cells length/width (Figure 7E), costa width (Figure 7F), number of cell columns forming the leaf border (Figure 7C), and number of differentiated perichaetial leaves (Figure 8D). The qualitative variables that contributed most to the separation of species were the twist direction of the upper seta, differentiation of perichaetial leaves (Figure 9N and Figure 10F,J), shape of the urn (Figure 9J and Figure 10C,H), costa ending (subpercurrent, percurrent, or excurrent) (Figure 9F–I and Figure 10I), and ornamentation and curvature of the upper leaf margins (Figure 9G,H and Figure 10I). It should also be noted that several of these morphotypes of *A. catillum* s.l can grow together on the same turf and with other *Aloina* species such as *A. brevirostris*, which further complicates their discrimination when specimens lack sporophytes.

Several of the morphological characters examined were congruent with the clades obtained in the molecular study and provide synapomorphies or combinations of character states that support the recognition of the three lineages in *A. catillum* s.l. (named A, B, and C in Figure 3, Figure 4 and Figure 5).

The study of the type material of available published names allowed the identification of the correct names for the specimens included in clade A. This study showed that the types of *A. catillum* and *A. calceolifolia* belong to the same species and, applying the principle of priority, the second name should be used. *Aloina calceolifolia* was described under *Tortula* [57] from a specimen collected by Spruce in the Ecuadorian Andes and was later transferred to *Barbula* Hedw. [58]. Finally, Brotherus [59] considered it as *Aloina*. Later, Delgadillo [13] included it among the synonyms of *A. rigida*.

The type material of *A. calceolifolia* studied from NY and E exhibits all the morphological characters of specimens grouped in clade A, although both plants and leaves (1.8 mm length) were slightly longer and the costa was more robust, with three layers of dorsal stereids. However, these characters are considered to be within the variation in the species. *Aloina calceolifolia* can be distinguished from the other species of *Aloina* with a leaf marginal border by its lingulate to suborbicular leaves, the leaf border developed only in the sheathing base (Figure 10A,B,E), margins usually strongly sinuose-papillose above (Figure 10B), and the leaf apex rounded to widely obtuse. Further, it has 1(2) inner perichaetial leaves usually differentiated, seta strongly twisted to the left throughout, a long cylindrical capsule (Figure 10C) [ratio urn length/width (3.3)3.9–6.6(7)] (Figure 8B)], spores 9.6–14.5 µm in diameter, and the usually monoicous condition.

Distinctive characters for specimens of lineage C ascribed to *A*. *catillum* are the ovoid-cylindrical urn (Figure 9J) [ratio urn length/width 2–2.8(4.2) (Figure 8B)], costa percurrent or excurrent as a mucro or awn, leaf margins strongly differentiated throughout the limb in 4–12 columns of cells (Figure 9D), and undifferentiated perichaetial leaves or occasionally with the innermost ones differentiated (Figure 9N). Consequently, it is here proposed as a new species, *Aloina limbata.*

The subclades C1–C4, defined by the molecular data, can be weakly recognized morphologically, with C1 being the best characterized. These C1 specimens have small, lingulate leaves (Figure 9C), with a narrow costa (100–150 µm wide at base) and 0–1(2) dorsal stereid layers. Although in the plastid tree these specimens are divided into two subclades C1a and C1b, no morphological differentiation has been found between them. The rest of the specimens of clade C included specimens with a tendency to have ovate to oblong-ovate leaves. The subclade C2, well supported in the ITS topology and split in the plastid tree, and clade C3 could not be morphologically delimited. The subclade C4 in the plastid tree includes the specimens identified as *A. obliquifolia* from the Southern Hemisphere (Figure 9A). In general, these specimens have the leaf border with many developed columns, the costa more strongly differentiated, with 2–3 dorsal stereid rows (Figure 9K), and usually excurrent as a mucro or stout awn (Figure 9F,H). The character of the development of an excurrent costa as a mucro or awn, which mainly defined *A. obliquifolia* [15,19], has been found here to be variable. Thus, it has been possible to find populations, even in the same plant, with a leaf costa percurrent, excurrent on a short mucro or into a stout awn. These South American *A. obliquifolia* can be easily differentiated from specimens of *A. obliquifolia* from the Northern Hemisphere (Figure 6D) because the seta is twisted to the left throughout and usually has developed papillae on the mucro or apex, a feature most readily seen in young leaves (Figure 9H).

Specimens of clade B are unambiguously distinguished from *A. calceolifolia* and *A. limbata* by 2–4 strongly differentiated perichaetial leaves (Figure 10J), ratio of upper laminal cell length/width 0.6–1 (Figure 7E) and the ovoid to elliptical capsule (Figure 10H) [ratio urn length/width 1.9–2.1 (Figure 8B)]. Other distinctive character states are the lingulate leaves, with strongly inflexed margins, subpercurrent costa, border differentiated from the sheathing base and part of the limb (Figure 10L), seta twisted to the left throughout, spores 13–16 µm in diameter, and operculum 0.6–0.8 mm long. These plants are here described as a new species, *Aloina bracteata.*

In the search for a name among those published that would fit these three lineages, we located the type material of *Barbula recurvipatula* Müll. Hal. (*Aloina recurvipatula*) at S (B4615). The specimen exhibits leaves with marginal border differentiated, only one shoot with undifferentiated perichaetial leaves, and a broken seta. Because it is difficult to identify it with confidence, we prefer to treat this taxon as one with doubtful identity.

## 4. Discussion

This study represents the first integrative approach to a group of *Aloina* species characterized by leaves bordered by hyaline cells. Based on our morphological and molecular analyses, we congruently identify the existence of hidden diversity in *A. catillum* and reveal the confusion that has existed among taxa identified as *A. brevirostris*, *A. catillum*, *A. obliquifolia*, and *A. rigida* in South America.

The existence of three well supported lineages in the phylogenies, which can be characterized morphologically within *Aloina catillum* s.l., indicates that this complex comprises three entities, recognized in this study at the species rank as *Aloina bracteata* sp. nov., *A. calceolifolia*, and *A. limbata* sp. nov. The ASAP delimitations inferred largely coincided with these clades based on the molecular phylogenetic results and morphological considerations.

According to our results, *A. calceolifolia* could be one of the basal groups of *Aloina*. It is very similar to some morphotypes of *A. limbata* because both species have similar leaf shapes, a rounded leaf apex, and the seta is sinistrorse throughout. In fact, specimens of *A. limbata* (Cano 223, 3083c, 3077b, 2125b, Cano and Gallego 3015, 3003b, Cano et al. 2814a, 2903a) and *A. calceolifolia* (Cano 2353, Cano 2176) were identified as *A. catillum* by Cano et al. [19]. Without sporophytes, these species are mainly distinguished by membranaceus leaf margins differentiated only in the sheathing base in *A. calceolifolia*. Since the neglected name *Aloina calceolifolia* is here resurrected, its distribution extends to dry areas of Argentina, Bolivia, and Peru.

*Aloina bracteata*, described here as a new species, forms a sister clade to *A. brevirostris* in the ITS tree and sister to *A. calceolifolia* in plastid topology. *Aloina brevirostris* as well as *A. bracteata* has a developed leaf border in the sheathing base and limb, lingulate leaves, and strongly inflexed margins. However, *A. brevirostris* usually has cylindrical capsules, and the seta turns to the right above. In addition, the perichaetial leaves are not strongly differentiated or have only the innermost ones slightly differentiated. *Aloina bracteata* is similar to *A. limbata*, but the former can be distinguished by smaller operculum and larger spores, longer upper and middle laminal cells, narrow leaf border (2–3 rows of cells), and 2–4 strongly differentiated perichaetial leaves. At the moment, it appears to be an endemic species to the Andes of northwestern Argentina (Salta and Tucumán).

*Aloina limbata* is a variable species in terms of leaf shape, width, and excurrence of the costa. In general, it is characterized by the strongly developed leaf margin in both the sheathing base and limb, seta twisted to the left throughout, and an ovoid-cylindrical capsule. Many of the *A. limbata* specimens studied lack sporophytes and, consequently, all sporophytic characters have not been properly analyzed. Molecular analysis and ASAP recognized some well-supported subclades. However, our morphological and molecular data suggest that boundaries are not yet fully formed. The only exception is the subclade C1, where molecular and morphological studies indicate that sympatric cryptic genetic variation may exist. It includes morphological specimens which are divided into two molecularly well-supported entities (C1a and C1b) in the plastid tree and in ASAP, but which do not differ phenotypically. The subclade C1 could possibly be recognized as a distinct variety or even species. However, thorough examination of a wider range of specimens, including samples with sporophytes, together with additional molecular sampling, will be necessary to confirm this. At present, we recognize the subclades as a potential example of ongoing speciation [60].

In the case of specimens identified as *A. obliquifolia* from the Southern Hemisphere, these can be easily distinguished morphologically from other specimens of this complex, when the mucro or awn is fully developed. However, this character has been found to be variable and our molecular analysis does not support this taxon as a Southern Hemisphere one. *Aloina obliquifolia* was reported from South America extending its distribution to the Southern Hemisphere, on the basis of two specimens collected in Ecuador and a Peruvian sample, all without sporophytes [19]. The American specimens shared with Northern Hemisphere specimens the leaf costa excurrent as a long mucro and the leaves bordered by thin-walled and hyaline cells. The study of new collections has revealed that the specimens identified as *A. obliquifolia* from the Southern Hemisphere have smaller spores, a smaller ratio between basal cells length/width, shorter upper laminal cells, narrower superficial ventral cells, more rows of marginal cells, and shorter leaves. Cano et al. [19] already pointed out some of these features, such as the smaller size of the leaves and more ovate leaves than in material from the Northern Hemisphere. The variability observed in the differentiation of the mucro in specimens identified as *A. obliquifolia* in South America has led us to consider these specimens within *A. limbata*. Further studies will determine whether these specimens merit any taxonomic rank. The results obtained with the two specimens of *A. obliquifolia* from the Northern Hemisphere included in the *A. brevirostris* clade in the molecular study, together with the high morphological variability observed in both sporophytic and gametophytic characters within *A. brevirostris*, also open the way for further studies.

Other results obtained in this work, such as the phylogenetic incongruence of nuclear and chloroplast DNA sequences in the positions of the accessions attributed to *A. rigida*-*A. brevirostris*-*A. ambigua*-*A. aloides* and, surprisingly, *A. bifrons*, may suggest incomplete lineage sorting, past hybridization, and historic introgression among populations in this group of species. This has already been indicated in other genera of the Pottiaceae [33,61,62] and calls for further future studies.

The tropical Andes are known as biodiversity hotspots and may be particularly conducive to cryptic speciation. The South American specimens we have studied, previously identified as *A. rigida*, are accepted here as *A. brevirostris* and *A. calceolifolia*. Consequently, *A. rigida* is tentatively excluded from this area and the distribution of *A. brevirostris* is enlarged to the Neotropics. After this study, seven species of *Aloina* have been confirmed in the area, five of them endemic. These results emphasize the need for integrative studies of numerous Pottiaceae species complexes in this area. Future fieldwork will be essential to complete the phylogeny of *Aloina* and to clarify the relationship in several cryptic or semi-cryptic complexes of this genus.

## 5. Taxonomy

Based on the morphological and phylogenetic analyses performed in the present study, we propose the following taxonomic treatment for the *Aloina catillum* complex. Complete descriptions, distribution data, and nomenclatural notes are also provided.

 

**Figure 11:**
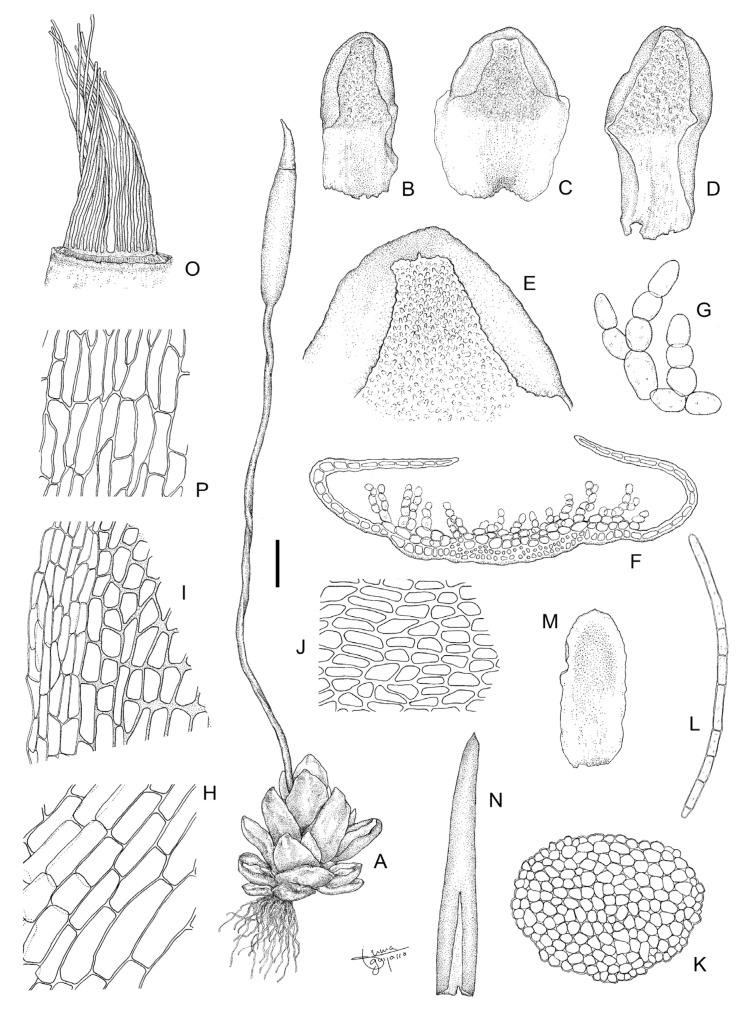
*Aloina calceolifolia* (Spruce) Broth. (**A**) Habit. (**B**–**D**) Vegetative leaves. (**E**) Leaf apex ventrally. (**F**) Cross-section of a leaf in middle part. (**G**) Detail of the ventral chlorophyllous filaments of the leaf. (**H**) Basal laminal cells. (**I**) Marginal laminal cells at base. (**J**) Upper laminal cells. (**K**) Cross-section of a stem. (**L**) Axillary hair. (**M**) Perichaetial leaf. (**N**) Calyptra. (**O**) Peristome. (**P**) Exothecial cells. Scale bars: A = 1 mm; B–D = 0.4 mm; E = 0.2 mm; F = 90 µm; G, H, I = 47 µm; J = 52 µm; K = 30 µm; L = 78 µm; M = 0.35 mm; N = 0.4 mm; O = 160 µm; P = 42 µm. (**A**,**O**,**P**) from Cano 2353 (MUB 20586); (**B**,**M**) from Cano et al. 3383 (MUB 27722); (**C**–**E**,**G**–**J**,**L**) from Cano and Alonso 8144 (MUB 56217); (**F**,**K**) from Cano et al. 4061c (MUB 61776); (**N**) from Cano et al. 7060 (MUB 48489). Illustration: Inmaculada Guijarro.

***Aloinacalceolifolia*** (Spruce ex Mitt.) Broth., Nat. Pflanzenfam. *in* Engl. & Prantl I(3): 428. 1902. *Tortula calceolifolia* Spruce ex Mitt., J. Linn. Soc., Bot. 12: 157. 1869. *Barbula calceolifolia* (Spruce ex Mitt.) A. Jaeger, Ber. Thätigk. St. Gallischen Naturwiss. Ges. 1871–72: 409. 1873. (Figure 1F, Figure 10A–F and Figure 11).

**Type citation**: “Andes Quitenses, Carguairazo (11,000 ped.), ad terram nigram aggrerum, *Spruce*, *n. 155*”. **Type**: Ecuador, Tungurahua, Andes Quitenses, Carguairazo [*Carihuairazo*], *Spruce* (Musci Amazonici et Andini nº 155) (lectotype, designated by Delgadillo [13], corrected here: NY!; isolectotypes: BM000720000–image!, E00165175!, G00265578–image!, G00113834–image!, PC0657869!).

*Aloina catillum* (Müll. Hal.) Broth., Nat. Pflanzenfam. *in* Engl. & Prantl I(3): 428. 1902. ≡ *Barbula catillum* Müll. Hal., Linnaea 42: 329. 1879. **Type citation:** “Argentinia subtropica, in montibus altioribus inter Siambon et Tafi. 1872”. **Type:** Argentina, Tucumán, Siambon-Tafi, 1872, *P.G. Lorentz s.n.* (lectotype, here designated: S B B3591! (P. Dusén’s moss herbarium); isolectotypes: H–BR 80004!, NY 01128073 (web)!, S B3592!, S B3593!, ***syn. nov.***

**Description:** Plants small, forming open turfs or gregarious, olive green to reddish or reddish brown. Stems 0.3–2(–2.3) cm high, simple or branched; in cross-section circular, hyalodermis, sclerodermis, and central strand absent; axillary hairs of 4–10 cells, all hyaline. Rhizoids densely covering stem, whitish to pale brown, smooth. Leaves incurved when dry, erect-patent to spreading when wet, broadly lingulate to suborbicular, (1.1–)1.4–1.8 × 0.6–1.1(–1–3) mm [ratio length/width (1.1–)1.5–2.1(–2.9)], not constricted, concave; lamina unistratose, not fragile, reddish in KOH; apex rounded to widely obtuse, not apiculate, cucullate; margins broadly incurved, often partially covering the leaf blade, parallel to the costa in both sides above, strongly sinuose-papillose in upper mid-leaf, bordered by (2)3–4 columns of hyaline, thin-walled, membranaceous cells differentiated only in the sheathing base, base oblong, with shoulders slightly marked, not decurrent; costa (250–)325–388(–450) µm wide at base, ending 2–5 cells below the apex, surface cells dorsally usually long-rectangular from apex to base, smooth, surface cells ventrally forming chlorophyllous, uniseriate, usually branched filaments, which extend to the lamina; in cross-section at mid-leaf broadly flattened, 35–53 guide cells in (1–)2–3 layers, ventral stereids absent, (1–)2(–3) rows of dorsal stereids, hydroids absent, surface cells dorsally slightly differentiated, ventral surface cells filaments disposed in 4–5(–6) layers of barrel-shaped, thin-walled cells, apical cell conical, rarely subglobose, (11–)21–28 × 11–13(–17.6) µm, usually smooth; upper laminal cells oblate-rectangular to oblate-fusiform, (5–)6.5–9.5(–13) × (16–)19–27(–40) µm [ratio length/width 0.2–0.5(–0.7)], thick-walled, not collenchymatous, smooth; middle laminal cells oblate-rectangular to oblate-fusiform, 5–9.5(–15) × (14–)16–29(–32) µm [ratio length/width (0.2–)0.3–0.8(–0.99], thick-walled, not collenchymatous, smooth; basal paracostal cells rectangular, (24–)32–61(–75) × 16–24 µm [ratio basal paracostal cells 1.4–3.8(–4.7)], thin-walled, collenchymatous, smooth; basal marginal cells rectangular, (24–)32–61(–75) × (6.5–)9.6–11(–13) µm [ratio length/width 1.5–5(–7.2)], thin-walled, smooth, hyaline. Structures of asexual reproduction absent. Monoicous (cladautoicous or synoicous), dioicous (?, when periquetia not seen). Perigonial leaves ovate to suborbicular, concave, 0.6–0.8 × 0.5–0.6 mm; perichaetial leaves usually differentiated from vegetative, mainly the inner, in number of 1(2), oblong-lingulate to spatulate, with plane margins, the most internal usually without dorsal costal filaments, 1.1–1.3(–1.6) × 0.4–0.7 mm [ratio length/width 1.9–2.7(–3.1)]. Setae 1 per perichaetium, 8–13.2 mm long, twisted to the left throughout, orange to brownish. Urns cylindrical, 2.1–3.8(–4.2) × 0.5–0.7 mm [ratio urn length/width (3.3–)3.9–6.6(–7)], reddish brown to yellowish brown; exothecial cells rectangular, rarely oblong-hexagonal, (31–)40–58 × 8–13 µm [ratio length/width 3.3–7], thin-walled; stomata phaneropores; annulus in 3–4 rows of vesiculose cells; peristome 16 split nearly to the base into two filiform teeth, 600–900 mm long, papillose-spiculose, straight or slightly twisted, orange, basal membrane 60–83 µm long; operculum conic, 0.8–1 mm long. Calyptrae cucullate, 2.7–3 mm long, yellowish brown, smooth. Spores spherical, (9.6–)11–13(–14.5) µm in diameter, pale brown, finely granulose.

**Habitat:** Banks, crevices of rocks and soils in puna, grassland, and interandean valleys; 2730–4215 m altitude.

**Distribution:** Argentina, Bolivia, Ecuador, and Peru. Endemic to tropical Andes.

**Nomenclatural note**: Delgadillo [13] considered material from NY as the holotype of *Tortula calceolifolia.* As this publication predates 1 January 2001, it should be considered an inadvertent lectotypification (Art. 7.11 and Art. 9.23, [63]) and is here corrected.

 

**Figure 12:**
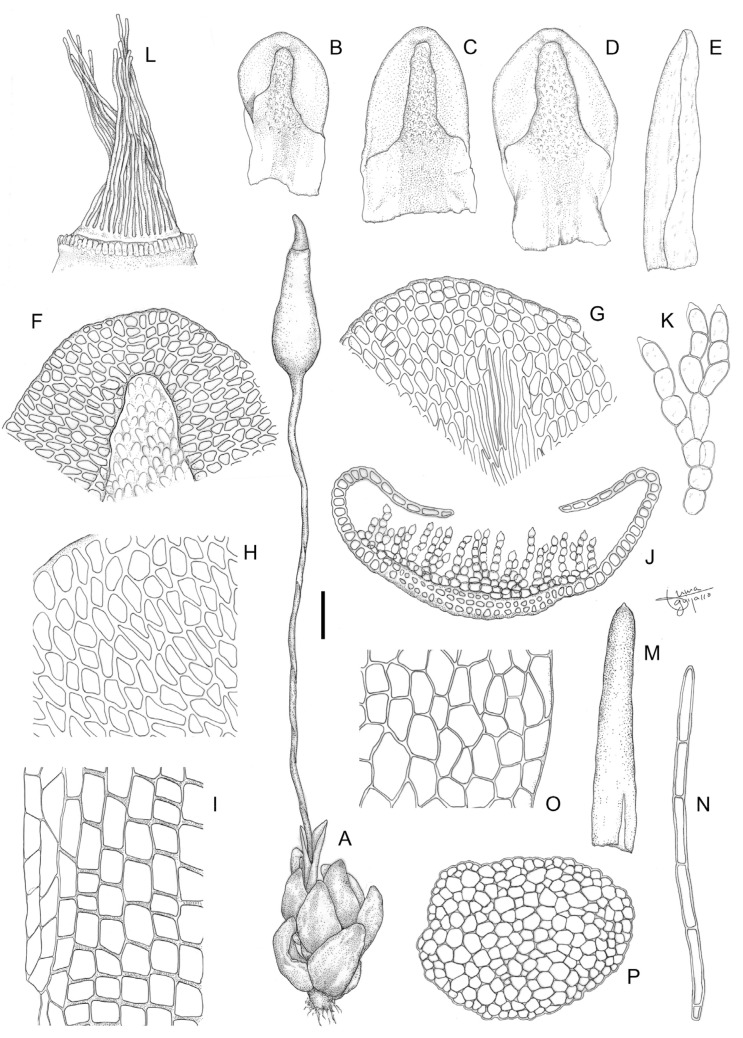
*Aloina bracteata* M.J. Cano et al. (**A**) Habit. (**B**–**D**) Vegetative leaves. (**E**) Perichaetial leaf. (**F**) Leaf apex ventrally. (**G**) Leaf apex dorsally. (**H**) Middle laminal cells. (**I**) Basal laminal cells. (**J**) Cross-section of a leaf in middle part. (**K**) Detail of the ventral chlorophyllous filaments of the leaf. (**L**) Peristome. (**M**) Calyptra. (**N**) Axillary hair. (**O**) Detail of exothecial cells. (**P**) Cross-section of a stem. Scale bars: A = 0.8 mm; B–D = 0.3 mm; E = 0.2 mm; F, G = 60 µm; H, I = 38 µm; J = 90 µm; K = 25 µm; L = 30 µm; M = 0.5 mm; N = 55 µm; O = 43 µm; P = 100 µm. (**A**–**P**) from Cano and Alonso 8409a (MUB 56491). Illustration: Inmaculada Guijarro.

***Aloina bracteata*** M.J. Cano, J.A. Jiménez & M.T. Gallego, **sp. nov.** (Figure 1D, Figure 10G–L and Figure 12).

**Diagnosis:** Differing from all other species of the genus *Aloina* by the following unique combination of character states: lingulate leaves, with strongly inflexed margins, border differentiated in the sheathing base and part of the limb, costa with two layers of dorsal stereids, ending below the apex, ovoid capsule, seta twisted to the left throughout, spores of 13–16 µm in diameter, operculum 0.6–0.8 mm long, and 2–4 perichaetial leaves strongly differentiated.

**Type:** Argentina, Salta, Iruya, 22°47′27″ N 65°13′26″ W, 2800 m, 27–III–2014, M.J. Cano and M. Alonso 8409a (holotype: MUB 56491!; isotype: CORD C0014154!).

**Etymology:** The specific epithet refers to the characteristic differentiated perichaetial leaves.

**Description:** Plants small, forming open turfs or gregarious, olive green to reddish or reddish brown. Stems 0.9–3 cm high, simple or branched; in cross-section circular, hyalodermis, sclerodermis, and central strand absent; axillary hairs of 7–10 cells, all hyaline. Rhizoids densely covering the stem, pale brown, smooth. Leaves erect, slightly incurved when dry, patent when wet, lingulate to obovate, occasionally oblong-lingulate or oblong-ovate, 1.2–1.5 × 0.7–1 mm (ratio length/width 1.6–1.8), not constricted, strongly concave; lamina unistratose, not fragile, reddish in KOH; apex rounded to obtuse, not apiculate, strongly cucullate; margins broadly inflexed, often partially covering the leaf blade, usually bent towards the costa in both sides in upper part, sinuose to dentate, bordered by 2–3 columns of hyaline, thin-walled, membranaceous cells from base to upper third, base oblong, with shoulders slightly marked, not decurrent; costa 250–413 µm wide at base, ending 2–11 cells below the apex, surface cells dorsally long-rectangular from apex to base, smooth, surface cells ventrally forming chlorophyllous, uniseriate, usually branched filaments, which extend to the lamina; in cross-section at mid-leaf broadly flattened, 28–40 guide cells in 1–2 layers, ventral stereids absent, 1–2 rows of dorsal stereids, hydroids absent, surface cells dorsally differentiated, ventral surface cells filaments disposed in (4–)5–6 barrel-shaped, thin-walled cells, apical cell conical, occasionally subglobose 16–23 × 8–13 µm, usually unipapillose; upper laminal cells rounded to quadrate, or transversely rectangular, 9.6–11 × 11–14(–19) µm (ratio length/width 0.6–1), thick-walled, not collenchymatous, smooth; middle laminal cells oblate-rectangular, occasionally quadrate, 6.5–13 × 9.5–13(–18) (ratio length/width 0.6–1), thick-walled, not collenchymatous; basal paracostal cells rectangular, 24–48 × 11–19 µm (ratio length/width 1.5–3.4), thin-walled, collenchymatous, smooth; basal marginal cells rectangular, 18–24 × 5–6.5(–10) µm, (ratio length/width 2–3.8), thin-walled, smooth, hyaline. Structures of asexual reproduction absent. Probably dioicous. Perigonial leaves not seen; perichaetial leaves differentiated from vegetative, in number of 2–4, oblong-lingulate, with plane margins and the most internal without dorsal costa filaments, 1.5–1.8 × 0.5–0.8 mm (ratio length/width 2.4–3.3). Setae 1 per perichaetium, 6.3–7.9 mm long, twisted to the left throughout, orange. Urns ovoid, occasionally elliptical, 1.5–2.1 × 0.8–1.1 mm (ratio urn length/width 1.9–2.1), pale brown; exothecial cells rectangular to quadrate-hexagonal, 24–67 × 17–30 µm (ratio length/width 1.6–2.1), thin-walled; stomata phaneropores; annulus in 3–4 rows of vesiculose cells; peristome of 16 split nearly to the base into two filiform teeth, 430–550 mm long, papillose-spiculose, straight, orange, basal membrane 20–32 µm long; operculum conic, 0.6–0.8 mm long. Calyptrae cucullate, 2.6–2.9 mm long, yellowish, smooth. Spores spherical, 13–16 µm in diameter, light brown, finely papillose.

**Habitat:** Banks in dry interandean valleys, growing with *A. brevirostris*; 2110–2800 m altitude.

**Distribution:** Argentina (Salta, Tucumán). Endemic to tropical Andes.

 

**Figure 13:**
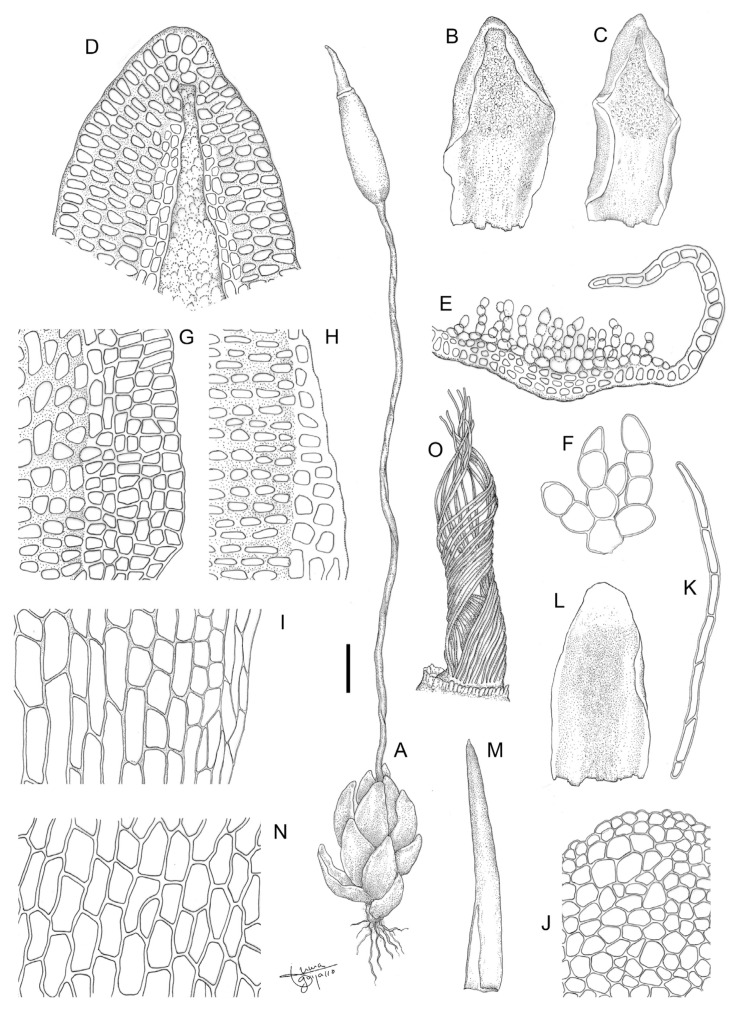
*Aloina limbata* M.J. Cano et al. (**A**) Habit. (**B**–**C**) Vegetative leaves. (**D**) Leaf apex ventrally. (**E**) Cross-section of a leaf in middle part. (**F**) Detail of the ventral chlorophyllous filaments of the leaf. (**G**) Middle laminal cells. (**H**) Upper laminal cells. (**I**) Basal laminal cells. (**J**) Cross-section of a stem. (**K**) Axillary hair. (**L**) Perichaetial leaf. (**M**) Calyptra. (**N**) Detail of exothecial cells. (**O**) Peristome. Scale bars: A = 0.9 mm; B–C, L = 0.35 mm; D–E, N = 53 µm; F–H = 35 µm; I, J = 40 µm; K = 50 µm; L = 30 µm; M = 0.5 mm; N = 0.5 mm; O = 100 µm. (**A**–**O**) from Cano et al. 5065 (MUB 32542). Illustration: Inmaculada Guijarro.

***Aloina limbata*** M.J. Cano, J.A. Jiménez & M.T. Gallego, **sp. nov.** (Figure 1C,E, Figure 9 and Figure 13).

**Diagnosis:** Differing from all other species of the genus *Aloina* by the following unique combination of character states: leaf border strongly differentiated in the sheathing base and most of the limb, costa with two layers of dorsal stereids, percurrent on excurrent as a mucro or awn, seta twisted to the left throughout, ovoid-cylindrical urn, and undifferentiated perichaetial leaves or occasionally with the most internal ones differentiated.

**Type:** Peru, Cajamarca, camino de Celendín, *pr*. Chinchín, 07°09′15″ S 78°24′54″ W, 3050 m, 16–VI–2009, M.J. Cano, J. Guerra, and J.A. Jiménez 5065 (holotype: MUB 32542!; isotypes: USM, S B332546!).

**Etymology:** The specific epithet refers to the strongly differentiated leaf border.

**Description:** Plants small, forming open turfs, olive green to reddish or reddish brown. Stems 0.3–1.9(–3) cm high, simple or branched; in cross-section circular, hyalodermis, sclerodermis, and central strand absent; axillary hairs of 5–8(–11) cells, all hyaline. Rhizoids covering the stem, pale brown, smooth. Leaves erect-incurved to incurved when dry, erect-patent to patent when wet, oblong-ovate to ovate or lingulate, 1–1.6(–2.5) × 0.5–1 mm [ratio length/width 1.3–2.4)], not constricted, concave; lamina unistratose, not fragile, reddish in KOH; apex rounded to obtuse, sometimes mucronate to long apiculate, cucullate; margins broadly incurved, often partially covering the leaf blade, parallel to the costa in both sides above, sinuose-crenulate to slightly crenulate, bordered by 4–9(–12) columns of hyaline, thin-walled, membranaceous cells from base to upper third or near apex, base with shoulders slightly or strongly marked, not decurrent; costa (113–)148–325(–432) µm wide at base, percurrent or excurrent as a mucro or awn, surface cells dorsally long-rectangular to linear, rectangular at base, smooth, surface cells ventrally forming chlorophyllous, uniseriate, usually branched filaments, which extend to the lamina; in cross-section at mid-leaf broadly flattened, 15–36(–40) guide cells in 1–2 layers, ventral stereids absent, 1–2(–3) rows of dorsal stereids, hydroids absent, surface cells dorsally slightly differentiated or undifferentiated, ventral surface cells filaments disposed in (4–)5–6 layers of barrel-shaped, thin-walled cells, apical cell conical to globose, (11–)14–24(–27) × (8–)9.5–13(–16) µm, smooth or unipapillose; upper laminal cells transversely rectangular to oblate or quadrate-rounded, (3–)5–9.5(–14) × (6–)9.5–21(–27) µm [ratio length/width 0.2–1.8(–2)], thick-walled, not collenchymatous, smooth; middle laminal cells transversely rectangular to oblate, (3–)5–8(–9.5) × (8–)9.5–21(–29) µm [ratio length/width 0.2–0.7(–1.2)], thin-walled, collenchymatous, smooth; basal paracostal cells rectangular, rarely quadrate, (11–)13–50(–75) × (5–)8–14.5(–19) µm [ratio length/width 0.8–3.7(–4.7)], 13–41(–75) × (5–)7–18 µm, thin-walled, collenchymatous, smooth; basal marginal cells rectangular, (11–)13–35(–40) × (5–)6.5–9.5(–13) µm [ratio length/width (0.8–)1.2–4(–6.7)], thin-walled, smooth, hyaline. Structures of asexual reproduction absent. Dioicous. Perigonial leaves ovate, strongly concave, ca. 1.4 × 1.8 mm; perichaetial leaves not or only inner scarcely differentiated from vegetatives, in number 0–1, oblong-ovate or lingulate, with flat margins and costa less developed, 0.9–1.8 × 0.4–0.7(–0.8) mm (ratio length/width 1.4–2.7). Setae 1 per perichaetium, 7.6–13(–15) mm long, twisted to the left throughout, reddish brown to pale brown. Urns ovoid-cylindrical, occasionally elliptical (1.1–)1.7–3.3 × 0.6–0.9 mm [ratio length/width 2–2.8(–4.2)], reddish brown to pale brown; exothecial cells rectangular, 23–53(–80) × 8–24 µm [ratio length/width 2–5.6(–8)], thin-walled; stomata phaneropores; annulus usually in three rows of vesiculose cells; peristome of 16 split nearly to the base into two filiform teeth, 420–900(–1250) mm long, papillose-spiculose, usually twisted one turn, yellowish brown, basal membrane 55–125 µm long; operculum conic, 0.8–1 mm long. Calyptrae cucullate, 2.2–2.7 mm long, yellowish, smooth. Spores spherical, 8–9.5(–11) µm in diameter, light brown, finely papillose.

**Habitat:** Banks, crevices of rocks and soils in puna, grassland, interandean valley, dry forests, and Chaco serrano formations; 2730–4215 m altitude.

**Distribution:** Argentina, Bolivia, Chile, Ecuador, Peru, and Venezuela. Endemic to South America.

## 6. Key to the South American Species of *Aloina* with Differentiated Leaf Marginal Border

1. Seta twisted to the right in the upper part, occasionally to the left below; monoicous (synoicous, or rhizoautoicous), rarely dioicous; leaves usually lingulate, more rarely suborbicular; spores 15–22 µm in diameter; central strand of the stem usually weakly differentiated .............................................................................................................................................................................................................................................. ***A. brevirostris***

1. Seta twisted to the left throughout; dioicous or monoicous (cladautoicous, synoicous); leaves lingulate to elliptical, oblong-ovate to suborbicular; spores 8–16 µm in diameter; central strand of the stem absent .......................................................................................................................................................... 2

 

2. Leaf border differentiated only in sheathing base; leaf margins strongly sinuose-papillose distally; urns usually cylindrical; usually monoicous ............................................................................................................................................................................................................................................ ***A. calceolifolia***

2. Leaf border differentiated from base to the upper third or near apex; leaf margins lightly crenulated to dentate; urns ovoid, elliptical to ovoid-cylindrical; usually dioicous .................................................................................................................................................................................................................. 3

 

3. Costa ending some cells below apex; 2–4 perichaetial leaves strongly differentiated from vegetative leaves; urns usually ovoid ................................................................................................................................................................................................................................................. ***A. bracteata***

3. Costa usually percurrent or excurrent as a mucro or awn; perichaetial leaves not or only the 0–1(–2) innermost ones scarcely differentiated; urns usually ovoid-cylindrical ....................................................................................................................................................................................................... ***A. limbata***

## Figures and Tables

**Table 1:** Statistics of the nuclear and chloroplast datasets analyzed in this study.

Locus	Number of Specimens	Newly Generated Sequences	Sequence Length	Parsimony Informative Characters
ITS	70	65	1012	204 (20%)
*atp*B**-***rbc*L	52	48	530	37 (7%)
*trn*G	55	50	34	34 (5.4%)
*trn*L-F	54	49	435	29 (6.7%)

## Data Availability

DNA sequences are available on GenBank and all authors agree with MDPI Research Data Policies.

## Appendix A

Vouchers and GenBank accession numbers for taxa used in the molecular phylogenetic analysis. Taxon name, country and next division, voucher (collector, number, and where the specimen is housed), and GenBank accession number for DNA sequences ITS, *trn*G, *atp*B-*rbc*L and *trn*L-F; a dash (−) indicates missing data. An asterisk (*) is provided for sequences retrieved directly from GenBank. Specimens of *A. limbata*, previously identified as *A. obliquifolia*, are indicated as *A. obliquifolia* SH (SH: Southern Hemisphere).

***Aloina aloides*** (W.D.J. Koch ex Schultz) Kindb.: 1185, Spain, Albacete, Gallego s.n. (MUB 56946), MZ239210, MZ290025, MZ339865, MZ290077. ***Aloina ambigua*** (Bruch & Schimp.) Limpr.: 1458, Cape Verde, Fogo, Cano 8114 (MUB 44972), PP002526, PP036083, PP036131, PP036033. ***Aloina bifrons*** (De Not.) Delgad.: 939, Spain, Madrid, Cano 6086 (MUB 34263), PP002523, PP036077, PP036124, PP036027; 985, Tunisia, Mahdia Governorate, Pocs et al. 03040/D (MUB 28451), PP002522, PP036078, PP036125, PP036028. ***Aloina bracteata*** M.J. Cano et al.: 937, Argentina, Salta, Cano and Alonso 8409a (MUB 56491), PP002524, PP036075, PP036122, PP036025; 1208, Argentina, Tucumán, Cano et al. 4113 (MUB 29597), PP002525, PP036076, PP036123, PP036026. ***Aloina brevirostris*** (Hook. & Grev.) Kindb.: 1300, Argentina, Catamarca, Cano et al. 4125 (MUB 29594), PP002535, PP036089, PP036137, PP036039; 1216, Bolivia, Oruro, Cano et al. 3403 (MUB 27728), PP002537, PP036088, PP036136, PP036038; 1465, Canada, British Columbia, Mclntosh NAD83 (UBC B239220), PP002536, PP036087, PP036135, PP036037; 1464, Canada, British Columbia, Bjork 22763 (UBC B227649), PP002532, PP036086, PP036134, PP036036; 1219, Russia, Krasnoyarsk Krai, Fedosov 17–2–15–08 (CAS 1288359), PP002534, PP036084, PP036132, PP036034; 1317, Russia, Krasnoyarsk Krai, Fedosov 16–0140 (CAS 1267137), PP002540, PP036092, PP036140, PP036042; 1196, United Kindom, England, Blockeel 47/384 (Herb. Blockeel), PP002533, PP036085, PP036133, PP036035; 1387, USA, New Mexico, Sargent 2591 (CAS 1290032), PP002539, PP036093, PP036141, PP036043; 1389, USA, New Mexico, Dillingham 2646 (CAS 1259598), PP002538, PP036091, PP036139, PP036041. ***Aloina calceolifolia*** (Müll. Hal.) Broth.: 1182, Argentina, Jujuy, Cano and Alonso 8345 (MUB 56422), PP002516, PP036071, PP036118, PP036021; 1836, Argentina, Salta, Cano et al. 8144 (MUB 56217), PP002513, PP036068, PP036115, PP036018; 1293, Argentina, Salta, Cano and Alonso 8168b (MUB 56242), PP002515, PP036072, PP036119, PP036022; 1838, Argentina, Tucumán, Cano et al. 4061c (MUB 61776), PP002521, –, –, –; 1478, Bolivia, Oruro, Cano et al. 3383 (MUB 27722), PP002519, PP036069, PP036116, PP036019; 1190, Peru, Ancash, Cano et al. 6879 (MUB 48303), PP002514, PP036073, PP036120, PP036023; 1451, Peru, Ancash, Cano et al. 7060 (MUB 48489), PP002518, PP036074, PP036121, PP036024; 1484, Peru, Arequipa, Cano 2176 (MUB 20394), PP002517, –, –, –; 1176, Peru, Puno, Cano 2353 (MUB 20586), PP002520, PP036070, PP036117, PP036020. ***Aloina limbata*** M.J. Cano et al.: 1304, Argentina, Catamarca, Cano et al. 4158 (MUB 28598), PP002490, PP036056, PP036106, PP036007; 1302, Argentina, Catamarca, Cano et al. 4267 (MUB 29589), PP002495, PP036050, PP036100, PP036001; 976, Argentina, La Rioja, Cano et al. 4324 (MUB 29599), PP002488, –, –, –; 1218, Argentina, Mendoza, Cano et al. 4374 (MUB 29595), PP002487, PP036052, PP036101, PP036002; 977, Argentina, Salta, Cano et al. 4082 (MUB 29585), PP002481, PP036046, PP036096, PP035997; 1294, Argentina, Salta, Cano and Alonso 8152 (MUB 56225), PP002491, PP036057, PP036107, PP036008; 1172, Argentina, Salta, Cano and Alonso 8205 (MUB 56282), PP002494, PP036054, PP036104, PP036005; 1180, Argentina, San Juan, Cano et al. 4350 (MUB 29587), PP002498, –, –, –; 1303, Argentina, Tucumán, Cano et al. 4085 (MUB 29592), PP002482, PP036047, PP036097, PP035998; 1306, Argentina, Tucumán, Cano et al. 4061a (MUB 29593), PP002500, PP036059, PP036108, PP036010; 1213, Bolivia, Chuquisaca, Churchill et al. 24638 (MUB 38330), PP002486, PP036051, PP036102, PP036003; 1207, Bolivia, Cochabamba, Cano et al. 3561 (MUB 27720), PP002483, PP036048, PP036098, PP035999; 1206, Bolivia, Cochabamba, Cano et al. 3565 (MUB 27725), PP002484, PP036049, PP036099, PP036000; 1476, Bolivia, Potosí, Cano and Jiménez 3699a (MUB 27723), PP002505, –, –, –; 1479, Ecuador, Azuay, Cano et al. 2814a (MUB 22313), PP002493, –, –, –; 1178, Ecuador, Azuay, Cano and Gallego 2903a (MUB 22314), PP002499, PP036060, PP036109, –; 1477, Ecuador, Loja, Cano and Gallego 3015 (MUB 22317), PP002479, –, –, –; 1191, Peru, Ancash, Cano et al. 7075 (MUB 48503), PP002489, PP036055, PP036105, PP036006; 1449, Peru, Cajamarca, Cano et al. 5157 (MUB 32544), PP002512, –, –, –; 1482, Peru, Cajamarca, Cano and Jiménez 5168 (MUB 32545), PP002480, PP036045, PP036095, PP035996; 1189, Peru, Huánuco, Cano et al. 7364a (MUB 48842), PP002506, PP036063, −, PP036013; 1485, Peru, Junín, Cano 2073a (MUB 20357), PP002503, –, –, –; 1480, Peru, Junín, Cano 2069b, (MUB 20581) PP002497, –, –, –; 1483, Peru, La Libertad, Cano and Jiménez 5357 (MUB 32546), PP002496, PP036058, –, PP036009; 1481, Peru, La Libertad, Cano and Jiménez 5373 (MUB 32547), PP002511, PP036067, PP036114, PP036017; 1214, Peru, Puno, Cano 2288b (MUB 23220), PP002478, –, –, –; 1205, Venezuela, Merida, Grande et al. 5938 (MUB 36828), PP002485, PP036053, PP036103, PP036004; 1452, Peru, Ancash, Cano et al. 7113 (MUB 48546), PP002492, –, –, –; 1209, Ecuador, Azuay, Cano et al. 2814b (MUB 59251), PP002504, –, –, –; 1210 (*A. obliquifolia* SH), Ecuador, Imbabura, Cano 3200b (MUB 22585), PP002510, PP036066, PP036113, PP036016; 1193, Peru, Ancash, Cano et al. 7150 (MUB 48588), PP002507, PP036065, PP036112, PP036015; 1212 (*A. obliquifolia* SH), Peru, Cajamarca, Cano et al. 5076b (MUB 32548), PP002502, PP036061, PP036110, PP036011; 556 (*A. obliquifolia* SH), Peru, Cajamarca, Cano and Jiménez 5196 (MUB 32549), PP002501, PP036062, PP036111, PP036012; 1292 (*A. obliquifolia* SH), Peru, Junín, Cano 2069a (MUB 20352), PP002508, PP036064, –, PP036014; 978 (*A. obliquifolia* SH), Peru, Lima, Cano et al. 7649 (MUB 49149), PP002509, –, –, –. ***Aloina obliquifolia*** (Müll. Hal.) Broth.: 1240, China, Yunnan, Wen Zhang and Shevock 16–7978 (CAS 1283278), PP002542, PP036094, PP036142, PP036044; 961, Japan, Nagano, Sato s.n. (MUB 52995), PP002541 PP036090, PP036138, PP036040. ***Aloina rigida*** (Hedw.) Limpr.: 1462, Cyprus, Limassol, Cano 10652 (MUB 59090), PP002531, –, –, –; 1275, Greece, Creta, Cano et al. 10367 (MUB 58849), PP002530, PP036082, PP036129, PP036032; 1459, Spain, Almería, Cano 9374 (MUB 51040), PP002529, PP036081, PP036128, PP036031; 1276, Spain, Córdoba, Cano 10285 (MUB 56743), PP002527, PP036080, PP036127, PP036030; 1453, Spain, Islas Canarias, Tenerife, Cano 8813 (MUB 49260), PP002528, PP036079, PP036126, PP036029; 125, Spain, Murcia, Cano 4839 (MUB 28930), MW398549, GU953707, PP036130, GU953732. ***Aloinella galeata*** var. ***andina*** (Delgad.) Delgad. & Schiavone: 274, Argentina, Catamarca, Cano et al. 4124 (MUB 29607), MW398550, JN968401, MW433018, JN968437. ***Crossidium squamiferum*** (Viv.) Jur.: 272, Spain, Canary Islands, Cano 4801 (MUB 28896), MW398558. JN968402, MW433022, JN968438. ***Erythrophyllopsis andina*** (Sull.) R.H. Zander: 86, Bolivia, La Paz, Cano and Jiménez 3825 (MUB 27961), MW398546, GU953698, MW433015, GU953723.
